# Medication-related adverse events in health care—what have we learned? A narrative overview of the current knowledge

**DOI:** 10.1007/s00228-021-03213-x

**Published:** 2021-10-06

**Authors:** O. Laatikainen, S. Sneck, M. Turpeinen

**Affiliations:** 1 Research Unit of Biomedicine and Medical Research Center Oulu, Oulu, Finland; 2 Department of Pharmacology and Toxicology, University of Oulu, Oulu, Finland; 3 Oulu University Hospital, Oulu, Finland

**Keywords:** Medication-related adverse events, Health care, Patient safety, Patient harm, Adverse drug events, Medication errors

## Abstract

**Purpose:**

Although medication-related adverse events (MRAEs) in health care are vastly studied, high heterogeneity in study results complicates the interpretations of the current situation. The main objective of this study was to form an up-to-date overview of the current knowledge of the prevalence, risk factors, and surveillance of MRAEs in health care.

**Methods:**

Electronic databases (PubMed, MEDLINE, Web of Science, and Scopus) were searched with applicable search terms to collect information on medication-related adverse events. In order to obtain an up-to-date view of MRAEs, only studies published after 2000 were accepted.

**Results:**

The prevalence rates of different MRAEs vary greatly between individual studies and meta-analyses. Study setting, patient population, and detection methods play an important role in determining detection rates, which should be regarded while interpreting the results. Medication-related adverse events are more common in elderly patients and patients with lowered liver or kidney function, polypharmacy, and a large number of additional comorbidities. However, the risk of MRAEs is also significantly increased by the use of high-risk medicines but also in certain care situations. Preventing MRAEs is important as it will decrease patient mortality and morbidity but also reduce costs and functional challenges related to them.

**Conclusions:**

Medication-related adverse events are highly common and have both immediate and long-term effects to patients and healthcare systems worldwide. Conclusive solutions for prevention of all medication-related harm are impossible to create. In the future, however, the development of efficient real-time detection methods can provide significant improvements for event prevention and forecasting.

## Introduction

During the twentieth century, several cases of severe medication-related adverse events (MRAEs) gained attention worldwide resulting in general understanding of the risk of patient harm presented every time medicines are administered. As a result, the structures of pharmacological care were revolutionized in all stages, from research and development to continuous surveillance and risk-analyses stretching the entire lifecycle of each medicine. Since then, medical care has been characterized by careful assessment of risk and benefit in order to ensure safe and effective medical care.

It is apparent that MRAEs cause different levels of patient harm, with the most serious events resulting in an increase in morbidity and mortality worldwide [1–3]. The effects of the less serious cases are often poorly detected but are, however, known to decrease medication compliance and result in suboptimal medical care among patients making the execution of cost-effective, rational medication difficult. Aside from this, MRAEs are connected to a variety economic and functional challenges that strain the healthcare systems [4].

Accordingly, research focusing on MRAEs has been ongoing actively in order to describe the issue and present potential solutions for improved prevention. Understanding the risk factors and mechanisms of MRAEs is also important in both developing medication processes within healthcare organizations but also safer care practices nationally and internationally. Although previous research in this field has been thorough and frequent, the general interpretations have been complicated by the high heterogeneity in event terminology, study settings, and methods used. Consequently, forming a clear overview of the issue can be hampered by comparing the vastly differing results of different studies, creating an obvious need for coherent and concise summary of the topic. The main objective of this narrative review was to collect recent information from studies conducted on the topic of MRAEs in health care, in order to describe the overall prevalence, risk factors, and surveillance methods currently used.

## Methods

**Fig. 1:**
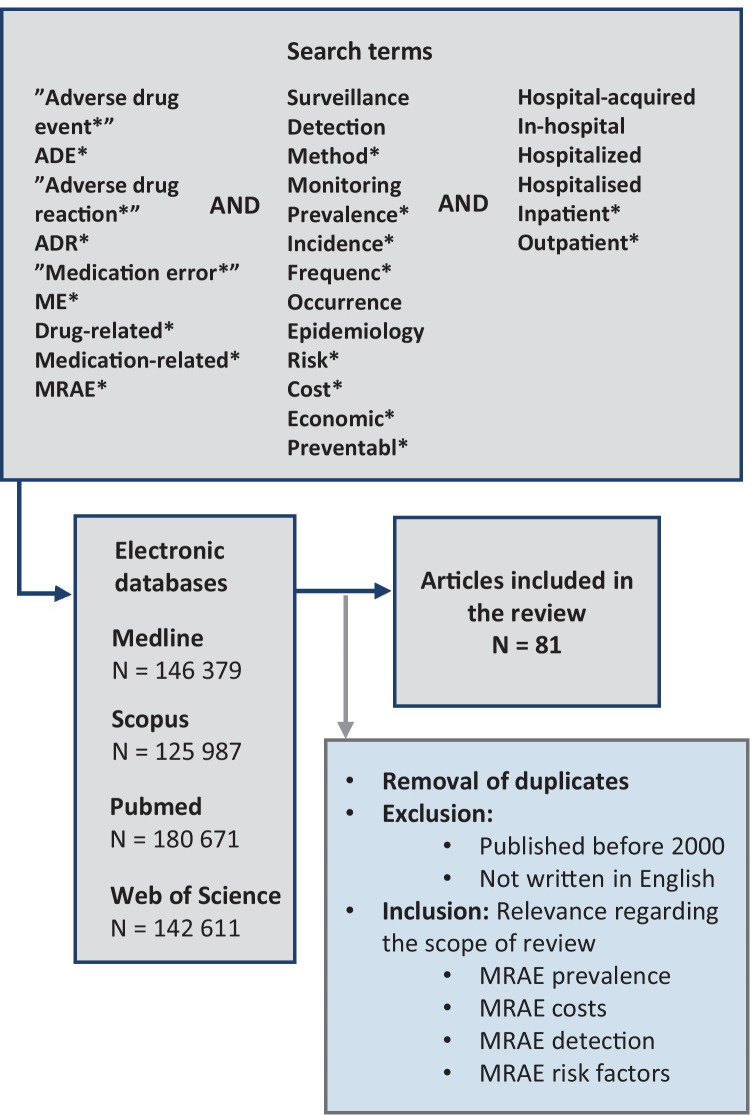
The database search method used in the narrative review

Electronic databases (PubMed, MEDLINE, Web of Science, and Scopus) were thoroughly searched with applicable search terms to detect relevant research on prevalence, risk factors, economic effects, and surveillance methods of medication-related adverse events. The search method and search terms are presented in Fig. 1. To form as up to date overview of the current situation as possible, only studies published after 2000 were included in this review. Systematic reviews and meta-analyses were preferred over other research, but high-quality papers describing original research were accepted as well. All articles that were not published in English were excluded. As this review was not conducted as a systematic review, information drawn from different articles was not filtered according to heterogeneity in terminology concerning MRAEs. This approach was selected as it also highlights the challenges associated with research concerning MRAEs. However, the differences in research terminology were acknowledged by reporting each drawn data as they were reported in the original article to avoid bias caused by mixed definitions.

## Definitions

**Fig. 2:**
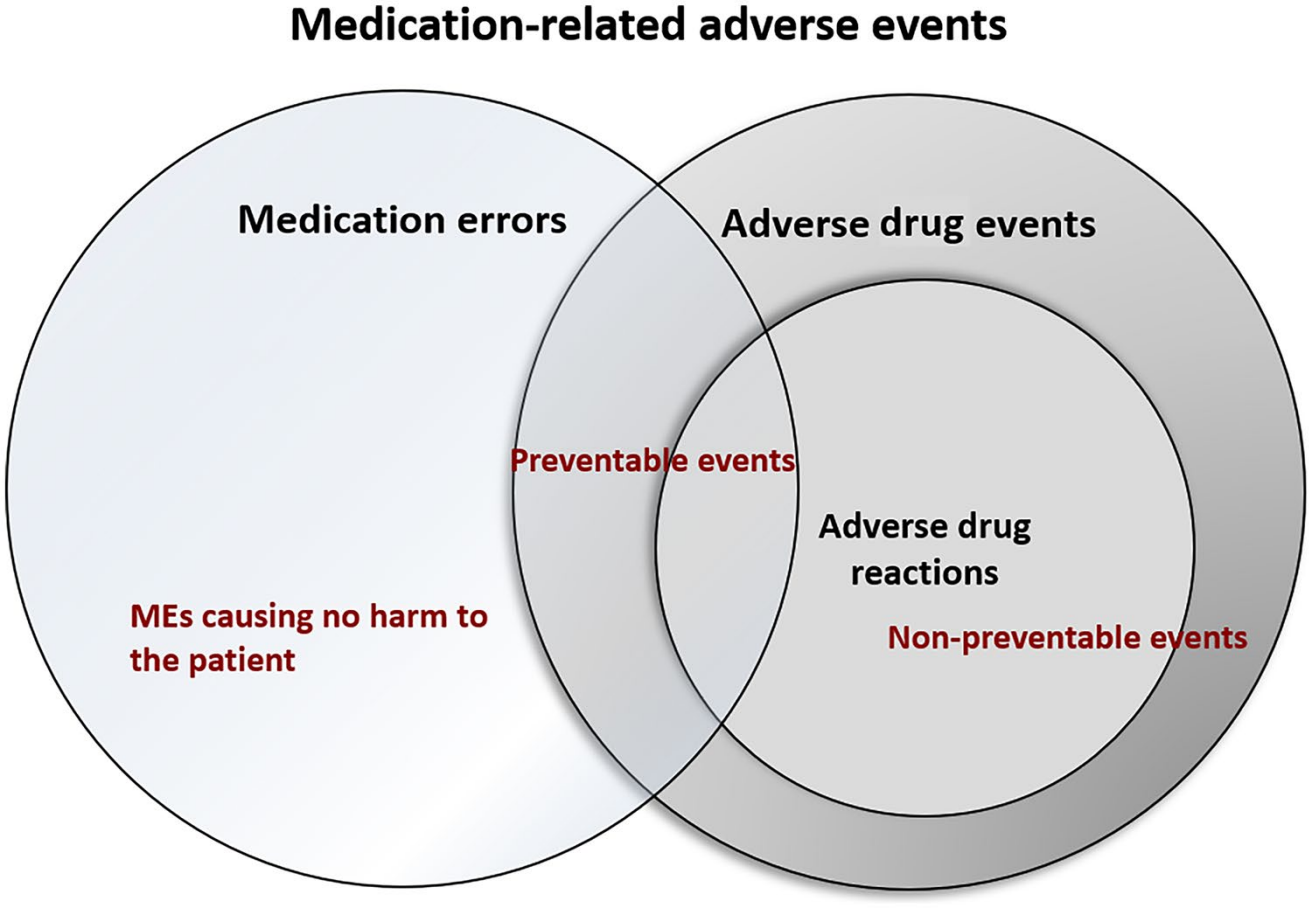
The interplay of different medication-related adverse events

In this study, ADE is defined according to the Institute of Medicine (IOM) as an injury resulting from medical intervention related to a drug and ADR according to the World Health Organization (WHO) as noxious or unintended response to a drug occurring at doses normally used in man [5, 6]. ME is defined by Ferner and Aronson as “a failure in the treatment process that leads to, or has the potential to lead to, patient harm,” and that can appear in any part of the medication process, e.g., in logistics, prescribing, handling, administering, or dispensing [7]. If not intercepted, MEs can lead to ADEs and ADRs. The term MRAE is used to describe all undesired events in pharmacotherapy, i.e., ADE, ADR, and MEs. The interplay of the terms is presented in Fig. 2.

## Prevalence of MRAEs

The number of studies focusing on all types of MRAEs has increased significantly over the past 30 years. Research has provided valuable insight into the frequency and nature of MRAEs but has also brought awareness of the heterogeneity between prevalence rates of individual studies (Table 1). Accordingly, depending on a variety of study characteristics, the prevalence of MEs varies anywhere from 0.8 errors per patient days to 22.2% of all administered medicines, and the prevalence of ADEs and ADRs from 12.9 ADEs per 1000 patient days to 58% of patients and 3.5 ADRs per 1000 emergency department (ED) visits to 14.7% of patients, respectively [8–13]. Similarly, high heterogeneity is seen in the results of various systematic reviews and meta-analyses conducted on this topic: in a meta-analysis by de Vries et al., the frequency of hospital-acquired ADEs was found 9.2% of all patients, but the numbers between meta-analyses differ up to 19% [14]. For in-hospital ADRs, the results of individual meta-analyses vary between 1.6 to 16.8% [15–17]. In a review by Keers et al., the overall prevalence for in-hospital MEs was found 19.6% [18]. Furthermore, as individual studies focusing on outpatient ADEs and ADRs causing unplanned hospitalizations to range between 2 and 5% in the overall population, a meta-analysis by Alhawassi et al. targeting unplanned hospitalizations of geriatric patients shows variation from 5 to 46%, with a mean prevalence of 11% [1, 4, 19–21]. Moreover, in a recent review, it was discovered that all MRAEs increase the risk of hospital readmission by a median of 21%, thus increasing the overall impact of in-hospital events [22].

**Table 1:** Variation in the prevalence rate of different MRAEs in various study settings. Adm = admission, pd = patient days

**Reference**	**Studied event (definition)**	**Study setting, study population**	**Prevalence %**
Cullen et al. [43]	ADEs (N/A)	Prospective study, ICU patients	19/1000 pd
Viana et al. [23]	ADEs (WHO)	Prospective study, all adult patients	24.7%
De Boer et al. [24]	ADE (Morimoto)	Observational multicenter cohort, surgical patients	15.4%
Dequito et al. [11]	pADE (An adverse event related to both a drug and a ME)	Prospective chart review, Patients from geriatric, internal medicine, and gastroenterology/rheumatology	58%
Kunac et al. [10]	ADE (actual injuries resulting from medical interventions)	Prospective study, pediatric patients	12.9/100 adm, 22.1/1000 pd
Davies et al. [12]	ADR (Edwards & Aronson)	Prospective study, internal medicine, and surgical patients	14.7%
Perrone et al. [13]	ADR (EMA)	Retrospective cohort study, emergency department patients, multicenter	3.5 ADRs/1000 ED visits
Giardina et al. [25]	ADR (WHO)	Prospective study, internal medicine patients	In 3.2% of patients during hospitalization, in 6.2% of patients the cause of hospitalization
Smith et al. [26]	ADR (N/A)	Retrospective analysis, neurosurgical ICU patients	10%
Choi et al. [8]	MEs (“any preventable event that occurs in the process of ordering or delivering medication, regardless whether an injury occurred or the potential for injury was present”)	Case–control study, voluntary error reports from selected hospitals	0.8/100 adm, 1.6/1000 pd
Härkänen et al. [9]	ME (“an incorrect dose, drug, delivery route, documentation, preparation, time, administration technique, administration of a defunct drug, or omission of a prescribed drug”	Prospective study, Surgical patients	22.2% of administered medicines
Kaushal et al. [27]	ME (“errors in drug ordering, transcribing, dispensing, administering, or monitoring”)	Prospective cohort study, pediatric patients	ME in 5.7% of prescriptions
Thompson et al. [28]	Medication-related problems (“an event or circumstance involving drug treatment that actually or potentially interferes with the patient experiencing an optimum outcome of medical care”)	Prospective study, obstetric and gynecological patients	201/241 patients with at least 1 medication-related problem
Urbina et al. [29]	Drug-related problems (“An event or circumstance involving drug therapy that actually or potentially interferes with desired health outcome”)	Prospective study, cardiology patients	29.8%

The main underlying reasons to the high heterogeneity in MRAE prevalence are largely due to differences in used definitions and study populations. Studies defining ADE and ADR in a way that includes events and reactions caused by misuse of drugs, e.g., abusing narcotics or using drugs to cause self-harm, and include more events than those excluding these types of situations through event definition. Differences may also arise from the interchangeable use of terms ADE and ADR, as well as incoherent association of MEs, ADEs, and ADRs [30]. In a mixed sample, detected numbers of MRAEs can differ substantially compared to studies focusing on specific risk groups [1, 31]. For example, ICU patients are known to be more susceptible to MRAEs with prevalence rates varying from 3.3 to 72.5% of patients whereas ME rates for psychiatric patients range from 10.6 to 17.5% [3, 32–34]. These interdisciplinary differences can largely be associated with both the frequency of medication use and the profile of commonly used medicines within organizations [33, 34].

### Economic consequences

MRAEs also have an important implication in healthcare economics and organizational functions [4, 8]. In this review, economic effects are presented as euros (exchange rate 1€ = 1.17 $US). Depending on the country or area, the total annual costs of all MRAEs can vary significantly. In the European union, the annual costs are estimated to reach €79 billion, whereas, in the USA, the estimates vary from €89.62 billion ($76.6 billion) to €207.56 billion ($177.4 billion) [35–38]. When different event types are singled out, estimates of in-hospital ME costs vary between €2.58 (unnecessary immunization in children) to €111 727 (litigation costs), and for in-hospital ADEs and ADRs, the costs range between €2647 and €7192 [4, 39–41]. When an ADE or ADR occurs in an outpatient setting and causes hospitalization, it is estimated to cause €6670 ($5700) excess costs [42].

Much of the variation between cost estimates is determined by the seriousness of the event, where minor events tend to cause smaller expenses and life-threatening events that require additional treatment and surveillance rapidly increasing the expenses [4]. Furthermore, care setting also plays important role in cost formation, and lower event-specific costs have been connected to primary care and non-intensive care compared to events in tertiary care and intensive care units [43]. Interestingly, it has been found that preventable events cause up to 1.8-fold higher costs than non-preventable ones [37]. Additionally, higher costs have been linked to certain specific symptoms, such as fever, bleeding, diarrhea, and cardiac arrhythmias [40]. Typically, the underlying reasons creating excess costs in MRAEs are increased length of stay, medication costs (e.g., additional treatments, exams, or medicines), and mortality [4, 12, 44]. From societal viewpoint, indirect costs are also created by indirect consequences, such as sick leaves and outpatient care making the total economic impact much greater than often first assessed [45].

## Known risk factors for MRAEs

**Fig. 3:**
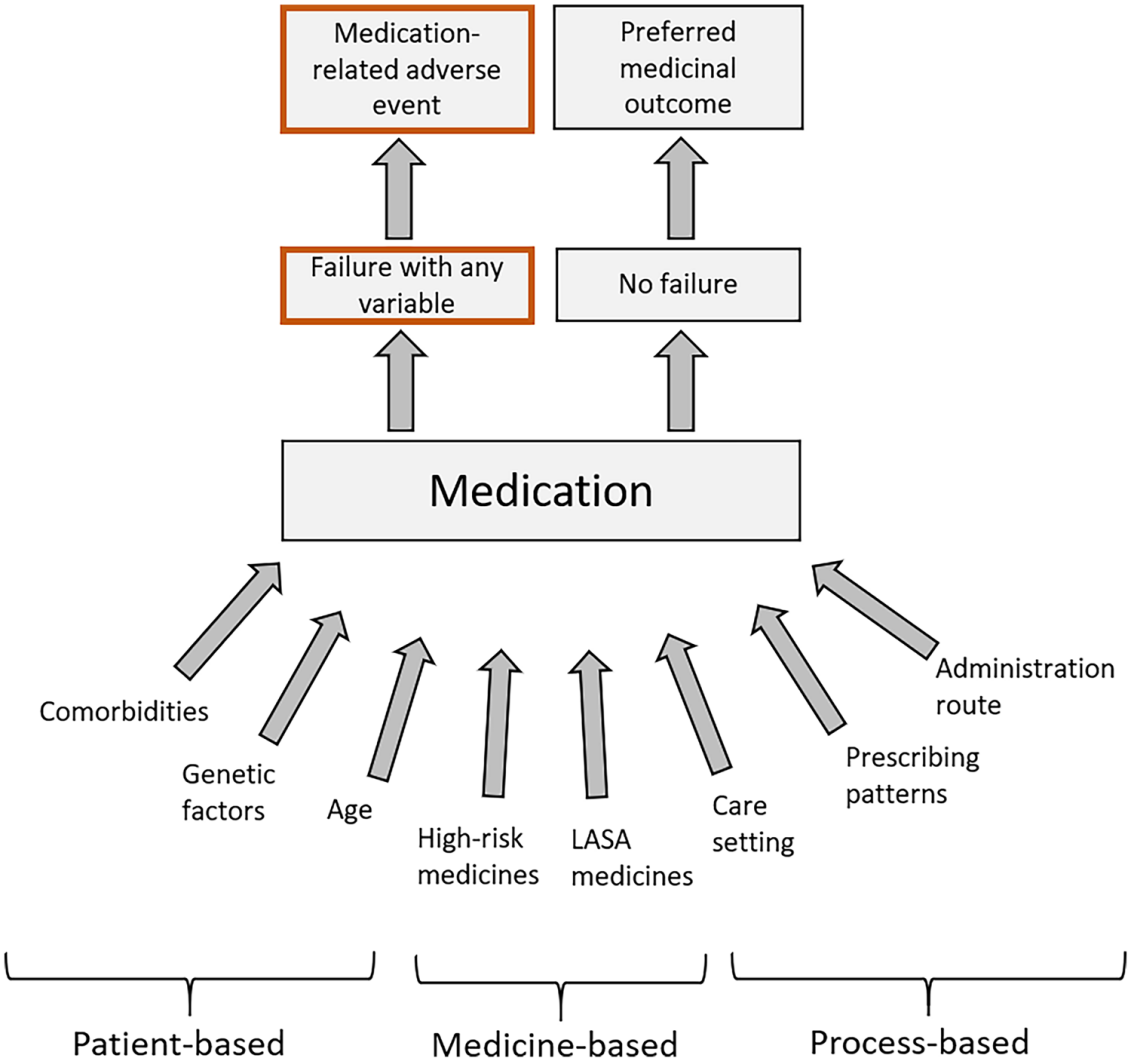
Variables affecting the different outcomes of pharmaceutical care

The effect of different variables on the medication outcome is presented in Fig. 3.

### Population-based risk factors

#### Genetic factors and age

Pharmacogenomics has shown that interindividual differences in the human genome can have a substantial effect to the ADME (absorption, distribution, metabolism, excretion) properties of medicines, causing inherent differences between individual patients in both therapeutic effects and the probability of ADEs [46–48]. However, several patient-based risk factors have also been detected, that are more generalizable to different patient groups at population level. Of these factors, studies have shown that higher age among patient has one of the strongest and most consistent evidence of increasing the susceptibility for MRAEs [1, 2, 39, 49–51]. Estimates show that MRAEs are up to 4 times more common in the elderly than in general population, in both inpatient and outpatient settings [1, 52]. As with current understanding, this results from multiple interconnected causes that occur with aging, including alternation in both pharmacodynamics (e.g., altered receptor function) and pharmacokinetics (lowered renal function, lowered metabolic capacity, changes in body mass distribution), increased complexity of comorbidities, and polypharmacy [1, 53, 54]. As all these factors would also independently increase the risk for MRAEs, it is obvious that the accumulation of them with aging is bound to cause problems, especially when combined with frailty and lowered capacity to sustain body homeostasis in the elderly. As a result, predicting the effects of even commonly used medicines can prove challenging in the elderly.

#### Multimorbidity

In addition to aging, changes in drug pharmacodynamics and pharmacokinetics can arise from other conditions in the general population. Multimorbidity can have a significant effect on therapeutic effect of a drug regardless of age [1, 49, 50, 54]. Similarly, polypharmacy, although most common in the aged population, will increase medication regimen complexity and predispose patients to MRAEs in all age groups. From individual conditions, decreased renal secretion and liver function have been identified as risk factors for MRAEs [2, 51, 55]. Of these conditions, decreased kidney function usually has greater clinical significance as it can rapidly alter the metabolism and disposition of drugs secreted mainly through the kidneys [46]. Although decrease in kidney function is a part of normal aging, especially quickly developing kidney diseases can have unexpected and severe consequences to pharmacological care. Hepatic dysfunction, on the other hand, will in most cases only have minor effects to drug disposition as its impact is often less straightforward. The influence can also vary even when drugs with the same metabolic pathway are taken, and the effects are also often complicated by their secondary effects on renal function [56].

Pediatric patients have been found more vulnerable to medication use due to their physiology and are more often affected by off-label use of medicines as a generally accepted practice than adults. However, pediatric patients consistently show lower numbers of medication-related hospitalizations than adults, ranging from 0.4 to 10.3% and a pooled estimate of 2.9% [57]. Similarly to adults, polypharmacy will increase the risk for MRAEs in pediatric patients as well [58].

### Medication-based risk factors

The risk for MRAEs is greatly influenced by the selection of drugs used in the treatment process [59]. The risk profiles of drugs can vary considerably, and a drug is typically considered “high-risk” when it has a narrow therapeutic range of reported history of verified severe ADEs [60]. The risk can also be heightened due to specific formulations, e.g., depot-preparations, intrathecal preparations, or care situations they are used in. Accordingly, especially errors conducted with high-risk medicines can cause severe consequences to the patient [61]. Different regulatory authorities and international organizations worldwide have constituted several lists for improved recognition of high-risk medicines.

In adult patients, such lists typically include medicine groups like anticoagulants, heparins, NSAIDs, antibacterials, diuretics, beta-blocking agents, chemotherapeutics, opioids, and psycholeptics [1, 32, 62–64]. In addition to the pharmacological and formulation-based problems, pharmaceutical preparations with higher risk for MRAEs through confusion over similar packaging or brand name or active substance name (look alike, sound-alike medicines) are also often considered in these listings [65, 66]. Although different variations of lists have been created for different care settings, e.g., acute care and long-term care, none of them is conclusive but rather directive, as they fail to consider many special features of different units and organizations. Thus, healthcare organizations are encouraged to create and uphold their own, individual lists for more accurate recognition of unit and function-specific high-risk medicines.

### Process-based risk factors

The medication process in healthcare organizations is a complex, multistage process including e.g., prescribing, transcribing, ordering, stocking, handling, preparing, dispensing, and administering medicines, demanding the joint participation of healthcare professionals from several different disciplines. Furthermore, as a patient is admitted to health care, the care process typically involves transmissions from one care setting to another, requiring fluent communication and information transfer to maintain proper medical care. In an in-hospital setting, medical care is often further complicated by the use of several invasive administration routes, that also increase the likelihood for MRAEs.

#### Prescribing

Previous studies show that MEs are most common during the administration and prescribing of medicines. Approximately 7% of all prescriptions are estimated to contain errors, typically resulting from inappropriate prescribing or prescribing a wrong dose [49, 66–68]. Inappropriate prescribing is, again, especially linked to the elderly, as it is estimated to affect 58% of geriatric patients and accounting for 25% of ADRs [66, 68]. Administration errors, on the other hand, are estimated to occur in 19.1% of total opportunities for errors, with approximately half of these errors occurring during the administration of intravenous drugs [69]. This is especially alarming, as administration stage errors are also estimated to be the error type least likely to be intercepted before affecting the patient, and thus, among all MEs, constitute the highest proportion of severe and fatal ADEs [70]. Other medication process stages commonly reported in MEs are transcribing errors (25.7%), dispensing errors (18.5%), and ordering errors (15.5%). MEs are, however, to a lesser extent, possible in all stages of medication process [71, 72].

#### Hospital admission

When the entire hospital admission period is considered, MRAEs have been detected especially frequently immediately after admission to the hospital as well as in care interphases. For example, Bobb et al. (2004) found more than half (64%) of MEs occurring at the time of admission [73]. This is further highlighted by latter findings, suggesting that 50% of MEs occur within 3 days of hospital admission and that major discrepancies in the majority of patients’ medication regimen tend to occur at the time of admission, placing the beginning of care particularly susceptible MRAEs [72]. However, discrepancies and other MRAEs are also shown to frequently occur in patient transitions and handoffs between care sites. In care interphase, the most common errors are the omission of drug from the patient’s medication regimen [74–76]. Care interphase and handoff errors have been tried to tackle with intervention by comprehensive medication reconciliation programs, with results showing a significant reduction in MRAE-related readmissions (67%), emergency department visits (28%), and hospital readmissions (19%) [77, 78].

## MRAE surveillance in health care

The detection and research focusing on MRAEs are a key element of medication safety: only active surveillance can provide means for prevention. Detection is also a fundamental component of pharmacovigilance and post-marketing surveillance, both of which have been proven necessary as only a small proportion of MRAEs occur during clinical trials. Although improvement leaps in this area have been made, to date MRAEs are known for being notoriously under detected and only approximately 3–10% of MRAEs are ever identified [40, 79, 80].

Currently, the surveillance of MRAEs relies on methods that can be categorized into 3 main classes: incident reporting, direct surveillance, and computerized methods [81, 82]. All 3 methods have shown great value in both research and in clinical implications. However, differences have been observed in event detection rates and types of events between different methods [82]. Furthermore, there is major variability in the clinical usability of each method. The qualities, strengths, and limitations of each method category are presented in Table 2.

**Table 2:** Main classes of detection methods for medication-related adverse events and most important advantages and disadvantages related to them. GTT = global trigger tool

**Method specifications**	**Incident reporting**	**Direct surveillance**	**Computerized monitoring**
Examples of used methods	Voluntary reporting Claims data	Chart reviews Patient interviews	GTT tool ICD-10 detection
Advantages	Reports in structured form Data is easily gathered Promotes culture of safety Cost-effective Easy to execute	Accurate Captures active errors and events Wide impact Good detection rates	Multidata-source integration Real-time method Enables ADE prevention Good detection rates Efficient use of electronic data on entire organization level
Limitations	Underreporting only allows retrospective analyses Variable quality of reports Blame culture	Costly Time-consuming Requires trained personnel for every unit where executed	Inserted errors Poor triggers Alert fatigue Requires expert assessment for actions to triggers

### Incident reporting

Incident reporting, mainly voluntary reporting, has been implemented for long in healthcare organizations worldwide [83]. Accordingly, it has held the status of primary detection method for MRAEs as it is both simple to execute and proven relatively cost-effective compared to e.g., direct surveillance which, although known for its thoroughness and good sensitivity, is laborious and expensive [84]. However, compared to the other two methods, incident reporting is undermined by significant underreporting, even when it is encouraged within healthcare personnel [85]. Furthermore, voluntary reporting has been found to mainly pick out latent errors with potentially harmful outcomes and events causing only minor harm thus leaving more serious types of MRAEs undetected [80, 83, 86]. Using voluntary reporting alone, it is not possible to form reliable estimates of the overall number of MRAEs.

### Direct surveillance and computerized methods

Compared to incident reporting, both direct surveillance and computerized methods can form better estimates of the overall prevalence of MRAEs, as both methods have shown better detection rates up to eightfold to that of voluntary reporting [81, 82, 84, 85]. Furthermore, as direct surveillance is usually conducted as real-time chart reviews or patient interviews and computerize methods as global trigger tool-type real-time alert-system, they both provide a clear advantage to incident reporting, which can only provide information for retrospective analyses. This enables faster responses to intervein and prevent serious harm related to MRAEs within healthcare units but also faster reactions to necessary changes on an organizational level. In direct surveillance, trained professionals are needed to be placed in every unit where surveillance is wanted, whereas one well-established trigger tool system can cover data from the entire organization. Nevertheless, it should be noted that trigger tools are only as good as the triggers they work by and that expert assessment of each alert is still required for selecting the appropriate actions for managing the situation [87]. Furthermore, direct surveillance remains the only reliable method for real-time detection of administration errors in the units and, without voluntary reporting, valuable information on inadequate processes and protocols resulting in MRAEs is inevitably lost. Thus, for the best possible coverage of MRAEs, variable combinations of each method category should be applied.

## Discussion

It is well established that MRAEs are a prevalent problem in health care, forming as severe of a health hazard in the western countries as malaria and tuberculosis do in the developing countries. Although MRAEs cause significant functional and economical challenges already, the impact of these events is estimated to further increase along with aging population and growing numbers of medication use all over the world. More than ever, research and patient safety work has focused on endeavors in active prevention of medication-related patient harm. It has given means to fight unwanted events by identifying risk factors related to medicines, processes, and patients in the medication process. However, we have also learned that simple solutions for eliminating the problem altogether are not available as the field is continuously changing and evolving. Thus, it is only through continuous research and method development that we can tackle and prevent medication-related problems as they arise.

All research is based on event detection and recognition. During the last decade, computerized methods have opened possibilities to answer to the growing demand of continuously increasing amount of electronic data in health care, combining different types of data sources, and automatizing information categorization. When combined with machine learning, computerized methods could provide further possibilities in event forecasting in all MRAE subtypes [88, 89]. This requires multidisciplinary strives from pharmacological, medical, and information technologies and could change the currently prevailing trend of preventative work through retrospective observations, also altering the nature of preventative work from trial and error to real-time actions and on-point prevention of patient harm. Such systems could also provide valuable information from other fields of medicine benefitting multiple functions within healthcare organizations, e.g., knowledge-based decision making and rational treatment [90, 91]. In the future, pharmacovigilance research and patient safety efforts should prioritize the supporting of collaborative research between computer science and medicine to create opportunities for the development of intelligent methods for preventative work.

There are several limitations to this article. As narrative review, this study does not provide a systematic and conclusive literature search of the topic. Therefore, it is possible that some factors related to the topics within this review are not reported here. Accordingly, this review does not provide any exhaustive quantification of medication safety issues presented here. However, in this article, it is possible to discuss the issues related to medical care without focusing on specific problem or variable and thus provides a comprehensive overview of the current status of medication-related problems in health care.
